# Particle Manipulation by Optical Forces in Microfluidic Devices

**DOI:** 10.3390/mi9050200

**Published:** 2018-04-24

**Authors:** Petra Paiè, Tommaso Zandrini, Rebeca Martínez Vázquez, Roberto Osellame, Francesca Bragheri

**Affiliations:** 1 Istituto di Fotonica e Nanotecnlogie IFN-CNR, Piazza Leonardo da Vinci 32, Milano 20133, Italy; petra.paie@polimi.it (P.P.); tommaso.zandrini@polimi.it (T.Z.); rebeca.martinez@polimi.it (R.M.V.); roberto.osellame@polimi.it (R.O.); 2 Dipartimento di Chimica, Materiali e Ingegneria Chimica “Giulio Natta”, Politecnico di Milano, Piazza Leonardo da Vinci 32, Milano 20133, Italy; 3 Dipartimento di Fisica, Politecnico di Milano, Piazza Leonardo da Vinci 32, Milano 20133, Italy

**Keywords:** optical manipulation, microfluidics, optofluidics, optical trap, optical tweezers, optical stretcher

## Abstract

Since the pioneering work of Ashkin and coworkers, back in 1970, optical manipulation gained an increasing interest among the scientific community. Indeed, the advantages and the possibilities of this technique are unsubtle, allowing for the manipulation of small particles with a broad spectrum of dimensions (nanometers to micrometers size), with no physical contact and without affecting the sample viability. Thus, optical manipulation rapidly found a large set of applications in different fields, such as cell biology, biophysics, and genetics. Moreover, large benefits followed the combination of optical manipulation and microfluidic channels, adding to optical manipulation the advantages of microfluidics, such as a continuous sample replacement and therefore high throughput and automatic sample processing. In this work, we will discuss the state of the art of these optofluidic devices, where optical manipulation is used in combination with microfluidic devices. We will distinguish on the optical method implemented and three main categories will be presented and explored: (i) a single highly focused beam used to manipulate the sample, (ii) one or more diverging beams imping on the sample, or (iii) evanescent wave based manipulation.

## 1. Introduction

Radiation pressure was first introduced by J. C. Maxwell in his theory of electromagnetism. It is the easiest and the most intuitive example of an optical force: light incident on a surface gives rise to a force on that surface. Being the intensity of optical forces rather small, from femto- to nano-Newtons, they are only effective on microscopic objects ranging from tens of nanometers to hundreds of micrometers. A real boost in the exploitation of optical forces to manipulate physical objects occurred with the invention of the optical tweezers by Ashkin and coworkers [1,2]. An optical tweezer exploits forces exerted by a strongly focused Gaussian laser beam to trap small objects. It can trap objects with dimensions ranging from 5 nm to 100 µm [3,4], and can exert forces up to 100 pN with fine resolutions [5,6,7,8,9]. This range is particularly interesting in the biological field since it corresponds to cells and organelles dimensions, hence to inter- and intra-cellular processes. The physical principles behind optical tweezers can be ascribed to different mechanisms whether the objects are much smaller or much larger than the wavelength of light. In the first case, the light’s electric field induces an electric dipole moment in the object that is pulled toward the focus by the intensity gradients of the electric field [10]. In the second case, Mie scattering conditions are satisfied and the problem can be solved by ray optics: larger objects act as lenses refracting the rays of light and modifying the momentum of photons, thus giving rise to recoil that draws the object towards the focus [11,12]. The optical force is usually described as the sum of two components: a ‘scattering force’, which pushes the particle along the propagation direction of the incident light, and a ‘gradient force’ that pulls the particle towards the highest intensity region and is due to the spatial intensity gradient. Stable trapping is obtained when the gradient force counterbalances the scattering force. To satisfy this condition, a steep spatial gradient of the beam intensity is needed, hence optical tweezers are usually realized by exploiting microscope objectives where high numerical apertures allow for focusing the light as tightly as possible [13].

Optical tweezers (OT) have been used for many diverse applications ranging from chemistry and physics to medicine and biology. In physical sciences, the capability of optical tweezers to manipulate matter in a non-invasive way allowed for studies in classical statistical mechanics, as, for example, measurements of macromolecular interactions in colloidal systems [14,15]. In medical and biological applications, optical tweezers have been exploited to characterize the forces exerted by molecular motors or, at the single cell level they have been used to study single cell mechanical properties by evaluating membrane elasticity. Moreover, they have been also exploited to probe viscoelastic properties of various samples, from single biopolymers as DNA to aggregated protein fibres [2,16,17]. Optical tweezers have been also exploited in areas, such as in vitro fertilization or in microsurgery to optoporate cells for chromosome and gene modifications [18,19,20].

Optical tweezers have been successfully used in many applications; also with the addition of different functionalities that have been implemented, e.g., sample rotation when beams with complex wavefronts are exploited [21]. Nevertheless, they still suffer from some limitations, the main being the serial nature of the analyses performed by tweezers that may limit the throughput and the amount of measured cells, which can be an issue in order to obtain statistically significant results.

In recent years, considerable effort has been devoted to the development of integrated optofluidic devices that are able to handle biological samples. Such devices usually rely on microfluidic circuits that perfectly match the need for single cell high throughput analysis by guaranteeing a controlled flow of the cells; optical radiations are then exploited to probe or manipulate the cells [22]. In fact, microfluidic systems allow for controlling the environment in which cells are manipulated, e.g., pH, temperature, drugs delivery, etc., so that in vitro experiments can be exploited to mimic in-vivo situations. Microfluidic Lab-on-chips (LOCs) usually consist of a network of channels with diameters ranging between 10–100 microns to transport very small volumes of biological samples, typically microliters to nanoliters. When the channel reaches the micrometer scale, inertia becomes negligible and viscous forces govern the flow [23,24]. The dimensionless Reynolds number (Re) gives an indication of the fluid regime, being the ratio between these forces; when Re << 2000 the fluid flow in a microfluidic channel is laminar, hence the mixing of two or more fluids with different properties is very slow since it is driven only by diffusion [25]. An important feature is that LOCs are usually made of transparent materials and can thus be easily coupled to microscopes in which optical tweezers are implemented. Optical trapping of latex beads in a laminar flow has been demonstrated in 1995 [26]; since then, optical tweezers in combination with LOCs have been used for many applications [27,28,29,30,31], resulting a powerful tool for the investigation of single-cell responses to environmental stimuli. 

More recently, different geometries of LOC have been implemented in order to realize automated devices to optically manipulate and analyse single cells with high-throughput so as to achieve statistically significant results. The trapping geometry based on two counter-propagating and non-focused laser beams has been extensively used to optically trap single particles and cells [32,33]. This dual beam laser trap is specifically suited for manipulating objects in a microfluidic environment: the laser beams, perpendicular to a microchannel, can be exploited to attract single cells from a flowing sample and hold them in place during analyses, such as imaging for size, morphology and structure inspection [34], or fluorescence and Raman spectroscopy [35]. As in the single beam case, the optical forces acting in this configuration can be divided in scattering and gradient force components. However, the particle will not be blocked, since the beam is not focused, but it will be pushed along the beam propagation direction, remaining in the maximum beam intensity region. Anyway, if the optical power of the two facing beams is balanced, the cell is stably trapped in the middle. In comparison to single beam optical tweezers, here, trapping is completely decoupled from the imaging optics and laser light intensities in the trapping region are two orders of magnitude lower. This is extremely important when cell viability has to be preserved to perform subsequent biochemical analysis. Non-focused laser traps have been exploited for the implementation of the optical stretching technique that allows for a reliable evaluation of single cells mechanical properties [36]. When the trapping optical power is increased a cell elongation is induced along the laser beam axis; by correlating the applied optical forces to the cell deformation the elastic modulus of the sample can be thus retrieved [37].

Optical forces can be exploited to manipulate objects even without directly impinging on them by exploiting evanescent waves that can be generated in correspondence to the interface between two media. This method can be particularly efficient when particles much smaller than the wavelength have to be trapped. The effects of evanescent waves were first investigated by Kawata and Sugiura [38]. They found that the particles were held on or close to the totally-internally-reflecting surface, and were transported along the surface by the transfer of momentum due to their scattered light. Since this first demonstration, many examples of particle manipulation through evanescent waves have been presented exploiting different optical elements as waveguides, optical resonators, or plasmonic structures. 

Independently from the optical manipulation method, the integration of optical traps in microfluidic environment allowed for obtaining microsystems capable of performing optical manipulation of single cells, as fluorescence analysis, mechanical probing, sorting, and collection, without damaging biological samples under investigations. In this paper we review significant examples of microfluidic devices exploiting optical forces to manipulate cells or particle: methods as single or dual beam laser trap and evanescent wave for optical manipulation are reported.

## 2. Optical Manipulation with Single Focused Beam

Since their first experimental demonstration, optical tweezers have obtained a significant impact in research laboratories, mainly because they offer an unprecedented tool for sample manipulation on the microscopic scale. From the very beginning, Ashkin and coworkers showed that OT could manipulate either living or inanimate biological material, and that with a proper choice of the trap wavelength the optical damage to the specimens under study was minimal [39]. During the last three decades optical tweezers have been further developed towards more versatile multifunctional tools by means of several technological refinements, like, for example, time-sharing approaches [40] or holographic beam-shaping [41], reinforcing their presence in modern laboratories. The microscopic scale of optical tweezers makes them particularly suitable to be integrated into microfluidic devices [42]. Moreover, the similarity in the technological background that is needed by an optical tweezer and microscopy techniques brings the opportunity to integrate them easily into lab-on-chip systems. In fact, the same microscope is used to implement the optical tweezer and to monitor a microscopic sample inside the microfluidic chip. Optical tweezers methods have found application in many diverse fields in lab-on-a-chip science encompassing microfluidic actuation and sorting, sensing the interaction force between microscopic objects in a microfluidic environment, and the characterization of micro-rheology properties. All of these applications will be briefly described in the next paragraphs focusing the attention on the role of the synergistic combination between optical tweezers and microfluidics.

The possibility to manipulate the specimens inside the microfluidic channels, independently from the fluid media and without physically “touching” them, opened up a broad portfolio of analyses that were otherwise not possible in a microfluidic device. For example, it was possible to freely move cells inside a complicated microfluidic network, keeping the flow unaltered and without contaminating the media. This was the idea of Hanstorp and co-workers in the early 2000’s when they used an optical tweezer in a microfluidic device for the first time [43,44]. They mainly exploited the unprecedented possibility to manipulate cells inside the microchannels independently from the sample flow. It consisted in an on-chip cell microculture system combining microfluidic cultivation chambers with optical tweezers, and the non-contact optical trap was used to transport cells between the cultivation, analysis, and waste microchambers. The independence between the medium flow and the cell manipulation enabled examining the effect of changes in nutrient condition on the growth and interdivision time changes of isolated single cells. The development of their pristine device lead to a complete Lab-on-a-chip, where, thanks to the optical tweezers, cells could be positioned on specific chambers without flowing (avoiding the contamination of the media), or they might be fixed while exchanging the media by microfluidic flow. The same chip allowed for performing microsurgery of cells, and, for the first time in a microfluidic chip, optical tweezers were used to trap and sort cells from an heterogeneous population [45]. These first “proof of concept” studies lead to more specific biological analysis moving cells between environments with different osmolarity, oxidative capacity, or glucose levels [46,47]. Chowdhury and co-workers studied a computational approach to speed up and increase the efficiency of cell transport between different growth chambers [48]. They followed a heuristic planning approach, which was based on D* Lite algorithm, introducing a novel state-action space representation and cost function in an attempt to obtain a completely automated system. They experimentally demonstrated it with a cell solution obtaining a success rate of six cells from 10 reaching the selected output of the chamber. 

### 2.1. Integration of Multiple Optical Tweezers

**Figure 1:**
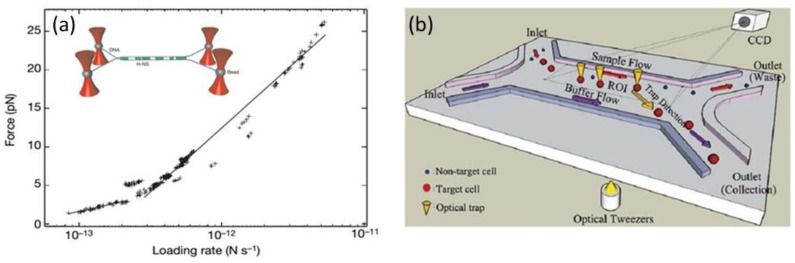
(**a**) Inset: Scheme of λ-DNA molecules linked by H-NS suspended between polystyrene beads held with optical tweezers; Dynamic force spectrum of the H-NS–DNA interaction. The two distinct regions in the Spectrum are fitted to straight lines. (Reprinted with permission from Springer Nature [50]); and, (**b**) Scheme of a lab-on-a-chip sorting procedure with optical tweezers (Reproduced from [51] with permission of the Royal Society of Chemistry).

The introduction of multiple optical tweezers increased the flexibility of the technique and consequently allowed for more complex analyses. One particular way of splitting a single trap into multiple traps is using a Holographic optical tweezer [49]. It consists in the addition of a computer controlled diffractive optical element that modifies the wavefront of the trapping laser (by a spatial modulation of the phase) and allows for splitting the single trap into multiple traps. Dame et al. used a quadruple trap to simultaneously control four microbeads attached to the ends of two DNA molecules, as it is represented in Figure 1a inset [50]. The independent handling of the two molecules allowed for the systematic investigation of molecule mediated DNA-DNA interaction and the evaluation of the binding force between DNA bridging proteins (see Figure 1a). These studies explained how the bacterial nucleoid can be effectively compacted and organized, being dynamic in nature and accessible to DNA-tracking motor enzymes.

Another example of force evaluation by optical tweezers is present in reference [52] where an array of holographic optical tweezers is used to create a microfluidic force sensor. They built a hexagonal scaffold of seven optically trapped polystyrene beads, loaded with fluorescently labeled actin filaments, which were then crosslinked via magnesium ions. During crosslinking the network contracts and the trapped beads follow this movement. As all of the beads were in calibrated optical traps, their movement could be directly related to the forces acting on them, obtaining a pN resolution.

### 2.2. Cell Sorting with Optical Tweezers

An immediate application of trapping and freely positioning cells inside microfluidic channels is to sort them depending on their specific properties. An example of a cell sorting chip is shown in Figure 1b [51], the sample and buffer are loaded separately into the device by two different inlets. A CCD camera is used to capture images of cells when they pass through the region of interest; target cells are recognized depending on their size and fluorescence by an image processing method. Afterwards, a control signal is generated to position the optical trap on the target cell and move it to the buffer flow. After the target cell arrives to the desired destination the trap releases it and it flows to the collection outlet where it will be collected and further analysed. With the use of multiple optical traps (five holographic optical tweezers) the sorting throughput was increased to a value of five particles per second. The sorter effectiveness was demonstrated by the purification of a mixture of Yeast cells with microbeads and a solution of fluorescent and non-fluorescent human embryonic stem cells (hesc). In the former case, they obtained a purity of 96% with a recovery rate of 94% while in the hesc solution they obtained a 90% purity solution with a recovery rate of 90%. In addition to holographic methods, there are examples of refractive multiple tweezers implemented by the use of an array of microlenses as in reference [53]. They developed a microfluidic array cytometer demonstrated by the stable trapping of more than 200 yeast cells in a microfluidic flow. Individual particles in the trapped array could be additionally picked, moved away, and be isolated from the main stream using a secondary steerable tweezer. Although the sorting throughput was low (2–3 particles per minute) they envisaged that it could be increased using an alternative method for steering tweezers. Landerberger et al. demonstrated an active optical sorter using a fast steerable optical tweezer [54]. Thanks to the simultaneous bright field image analysis of the flowing cells and the dynamical optical trapping, they implemented a self-optimization process that allowed for sorting different cells depending on their size and shape. With their device the sorting of yeast cell resulted in a recovery rate of 95% with a throughput of 1.4 cells/s. In the work of Ma et al., it is demonstrated that with two conventional optical traps, with different wavelengths and intensity profiles, it is possible to sort between particles having different sizes. The two optical tweezers are patterned in a ‘Y’ configuration, which is capable of effectively transporting and sorting yeast cells, differing in size, within a microfluidic chip [55].

A sorting step was also needed by Mirsaidov et al. to select the cells that were later used to synthesize tissue in the same microfluidic chip [56]. They used time sharing holographic optical tweezers to organize the cells into a complex array, which was afterwards encapsulated in a micro-sized volume of a photo-polymerizable hydrogel that mimics an extracellular matrix. By a step and repeat method, they extended the size of the encapsulated-cells volume until they obtained a super array of 200 cells. They created a heterogeneous array of *E. coli*, which were genetically engineered with a lac switch functionally linked to fluorescence reporter in order to monitor the viability and metabolic activity during the whole tissue synthesis.

An interesting example of a single optical tweezer used for sorting of cells is the one that is presented by Li et al. where they used the same femtosecond laser to fabricate the microfluidic chip (by water assisted surface ablation), and afterwards to perform the optical tweezer [57]. They presented a microfluidic device composed of two micropools in which they manipulated and sorted B substilis cells.

### 2.3. Fluid Control Actuated by Optical Tweezers

In addition to allow particle trapping and positioning, with optical tweezers it is also possible to drive fluid control within microfluidic channels by the rotation of dedicated microscopic structures. This is accomplished either by spatially rotating the tweezer beam, by choosing a particular shape of the rotating micro-structure (fabricated with the help of laser photopolymerization) or by configuring the phase and the polarization of the tweezer beam. Terray et al. used the scanning laser optical trapping method [58] to implement microfluidic gears or peristaltic pumps [59].

**Figure 2:**
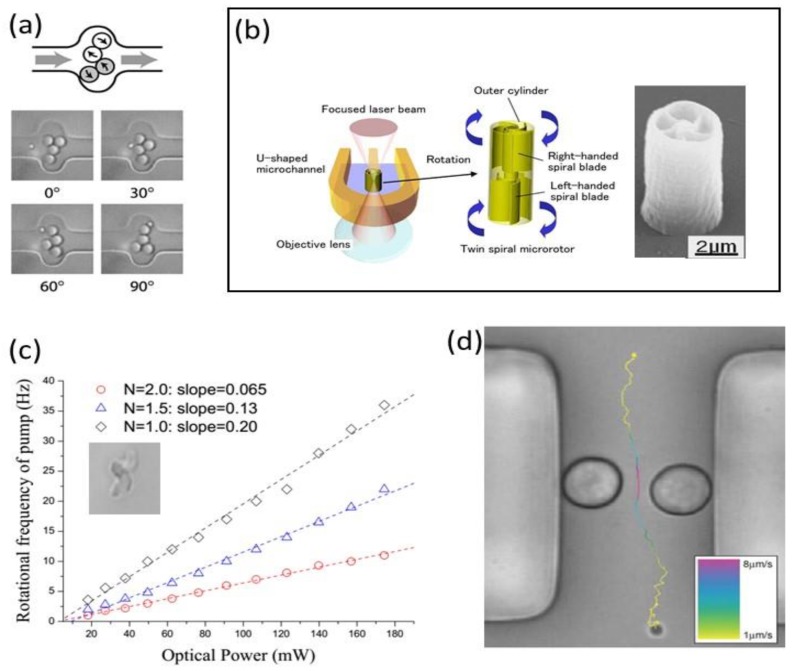
(**a**) Design of the gear microfluidic pump showing bead movement together with the screen shoots of 3 µm silica beads 2 Hz rotation in a 6 µm channel (From [59]. Reprinted with permission from AAAS); (**b**) Design of a micropump with the twin spiral microrotor, there’s also shown the Scanning electron microscope image of the polymerized microrotor (From [60]. Reproduced with permission of OSA); (**c**) Dependence of rotational frequency with trap power for Archimedes micropumps with different numbers (N), in the inset there’s a microscope image of a polymerized microscrew (height 5 µm) (From [61]. Reproduced with permission of OSA); (**d**) The traced path of a 1 µm silica particle being pumped through a 15 µm wide PDMS channel by the two trapped Vaterite beads. The colour of the trace changes as the particle accelerates and then decelerates through the channel due to the pumping effect (Reproduced from [62] with permission of de Royal Society of Chemistry).

To actuate the fluid multiple micro-size silica particles were moved by steering the tweezing beam through a scanning mirror. As it is reported in Figure 2a, for the gear pump, pairs of particles are rotated in opposite directions inducing a net flow. For the peristaltic pump, the particles are moved forward and backwards in a cooperative mode to obtain fluid propagation.

They also implemented a microfluidic valve, but this time exploiting laser photo-polymerization to organize silica beads in a specific geometry [63]. The micro-structure consisted on a silica bead (3 µm diameter) fixed to a row of smaller silica beads (0.65 µm), and the constructed passive valve was then actuated by the same laser used for the polymerization. The possibility to use two photon polymerization to manufacture micro-tools with arbitrary three-dimensional (3D) geometries has been used by other research groups again with the scope of actuating the fluid inside the microchannel. Maruo et al. created a spinning micro-rotor with twin spiral blades [60] that works as a microfluidic micropump that is driven by a focused laser beam, as reported in Figure 2b. The pump is positioned in a U shape channel and the optical tweezer has a double purpose; first, it maintains the pump on the desired position, and second, it induces an optical torque on the spiral blades that makes them rotate on the same direction reaching rotation speeds as high as 500 rpm. With a similar approach Lin et al. created Archimedes micro-screws (see Figure 2c inset) and performed a complete characterization of their rotating properties when actuated by an optical tweezer, also demonstrating their application as optofludic micro-pump [61]. In Figure 2c, the expected rotational frequencies of different pumps depending on the number of screws and the laser power are reported, demonstrating the linear dependence of rotating frequency with tweezer power. They demonstrated a microfluidic flow rate of 6 pL/min using a 1-screw micro-pump and a laser power of 200 mW (that induces a rotating frequency of 40 Hz). An elegant alternative to these strategies was explored by Leach and co-workers [62]. They exploited the spin momentum transfer from a circularly polarized tweezer laser beam to birefringent trapped particles. In particular, they used Vaterite micro-sized spheres with diameters ranging from 5 µm to 7 µm. As shown in Figure 2d, they placed two particles on opposite sides of the channels and next to the walls, the inverted handedness of the trapping beams induced an opposite rotation of the Vaterite particles and as a consequence a net fluid flow. With this device they demonstrated flows up to 200 fL/s with the two birefringent particles rotating at frequencies of 8.7 Hz and 9.2 Hz, respectively.

### 2.4. Micro-Rheology with Integrated Optical Tweezers

The actuation of microparticles inside microfluidic channels can also be used to mechanically characterize the fluid medium from a rheological point of view. Indeed, having a complete knowledge of the fluid viscosity is a priority in most microfluidic experiments, since the flow and mixing inside microfluidic channels depends on fluid viscosity. These experiments are based on the study of the motion of a trapped particle inside the microchannel, as it directly depends on the forces that are acting on it. Once the forces exerted on the micro-particles by the optical tweezer are known, it is possible to retrieve shear forces due to the fluid medium and, consequently, the medium viscosity with a microscopic accuracy [64]. Here, we are just going into the experiments where these measurements were done in a microfluidic chip; for a deep insight into general micro-rheology with optical tweezers, there are an interesting critical review by Yao et al. [65] and a recent book edited by Tassieri [66]. By the use of holographic optical tweezers, and with the help of a fast CMOS camera, Keen et al. correlated the thermal motion of multiple trapped silica micro-spheres with the viscosity at different points, and probed local changes in viscosity due to the presence of boundaries (as i.e., microchannel walls) [67]. In alternative, as for fluid control experiments, the laser scanning optical system has been used in micro-rheology experiments. Zhan et al. used a continuous steering along the optical axis of an optical tweezer to create a continuous circular translation of trapped micro-sized PMMA particles [68]. They developed a method to relate the particle escape velocity with the viscosity of the surrounding medium that is applicable either to Newtonian or non-Newtonian fluids. The final device was demonstrated measuring the viscosity of ethanol, FBS serum additive, and different DNA solutions.

From the previous discussions, it is evident that the use of optical tweezers is limited to systems based on microscopes, nevertheless new technological approaches are becoming available that will allow for the use of optical forces to implement the control, driving, and sensing in totally integrated lab-on-a-chip systems. For example, Liberale et al. [69] implemented a totally integrated optical tweezer based on an optical fiber approach. By two photon polymerization, they fabricated an optical micro-prism on the tip of a fiber in order to deflect the output beam. They obtained a stable optical trap that allowed for them to perform fluorescence and Raman measurements on biological cells.

Alternative new approaches to integrate optical trapping in lab-on-a-chip will be discussed in the next two sections.

## 3. Diverging Beam Particles Manipulation

**Figure 3:**
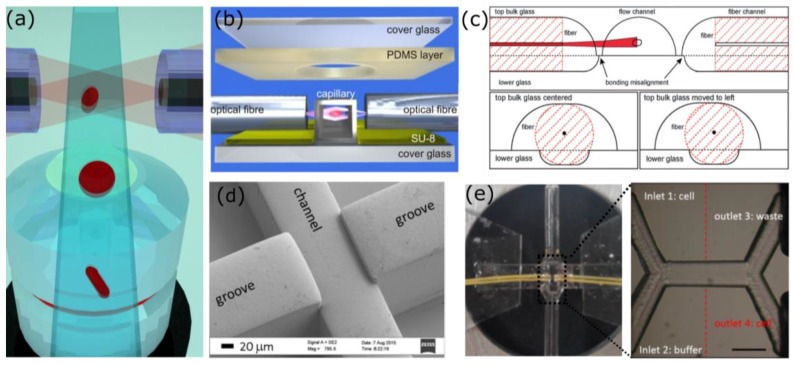
Panel (**a**) shows a scheme of the dual beam optical manipulation approach, with optical fibers used to deliver light to the microfluidic channel. Panels (**b**) to (**e**) show different devices for particles trapping and stretching. In panel b the scheme of the Assembled Optical Stretcher is reported (image reproduced with permission from [70]). Panel (**c**) shows the design proposed by Faigle et al., constituted by two asymmetrically etched substrates [71], published by the Royal Society of Chemistry. Panel (**d**) shows the disposable version of the Optical Stretcher (OS) proposed by Matteucci et al. (reproduced from [72]). Panel (**e**) reports the picture and the microscope image of the monolithic device presented by Yang et al. fabricated by FLICE (reproduced from [73] with permission from the Royal Society of Chemistry). Red traced lines indicate the waveguides position, scale bar is 100 µm.

An approach that has been largely used so far for particles manipulation in a microfluidic environment, foresees the use of two diverging light beams, which can be delivered using optical fibers, as schematically shown in Figure 3a. With this approach, it has been demonstrated that it is possible to trap particles, and also to stretch, to rotate, and separate them. With respect to particles manipulation by means of a single highly focused beam, this approach presents several advantages: an easy fiber-based on chip light delivery, a reduced risk of photodamaging the sample, which preferentially occurs with focused light beams, and also the possibility to integrate optical investigation techniques to retrieve simultaneously complementary data on the same sample with different approaches.

### 3.1. Particles Trapping and Stretching

The dual beam optical trap was first realized by Ashkin back in 1970 [74], 15 years before the first optical tweezer. Subsequently, the first fiber-based version of the optical trap was successfully used to trap polystyrene spheres and yeast cells in 1993 by Constable et al. [32], and several interesting declinations of this first fiber-based approach followed the first demonstration [75,76]. In 2006, Jess et al. proved the possibility to efficiently combine a dual beam optical trap and Raman spectroscopy with a microfluidic approach, to serially stop the particles and to analyse them by means of a label free discrimination approach, polystyrene particles and HL60 were successfully analysed [34]. An important milestone of the diverging beam based optical manipulation was demonstrated by Guck et al. in 2000 [36,77] who presented the Optical Stretcher (OS), which is an innovative approach to investigate the mechanical properties of cells. Indeed, by simply raising the laser power of a stable dual beam optical trap, the specimens are elongated along the beams propagation directions and the deformation can be measured and analyzed using a microscope and a CCD camera. The OS is a tool of high interest considering that cells mechanical properties are a valuable label free marker for functional changes in the sample, such as cancer progression, differentiation, cell cycles, and also infections [78,79,80]. It is interesting to stress that this non-contact approach compares favourably to other well-established methods used to measure cells mechanical properties, such as micropipette aspirations [81] and atomic force microscope [82,83]. This is due not only to the lack of mechanical contacts that can affect cells viability but also to the throughput, especially when considering that up to 10 cells/minute can be processed with the OS, while only about 10 cells/hour are analyzed with the other approaches.

The realization of a stable optical trap but even more of an efficient and reliable OS device is not effortless; therefore, several versions were presented in literature, where different fabrication approaches have been used to address the technical challenges that have to be satisfied to guarantee repeatable data [84]. Indeed, it is of crucial importance to have the two fibers equally distant from the channel and perfectly aligned one to the other. But also, the distance between the end of the fiber and the center of the channel, where the trap occurs, has to be finely optimized to maximize the trapping stability and the stretching efficiency, since it affects the dimension of the beam imping on the sample. These technical challenges highly impact the device efficiency, pushing for a technology capable to fully address these issues. The first fiber based optical traps, as well as the first OS [32,36,85], were realized using discrete elements: two opposite fibers that symmetrically face a sample chamber. In spite of the simplicity of this method, mechanical vibrations and instabilities revealed this approach as very impractical and with low experimental repeatability. Therefore, the subsequent versions tended to realize integrated devices, creating compact platforms that included both optical and fluidic elements. A first integrated version, the Assembled Optical Stretcher (AOS), was obtained exploiting the fabrication of grooves in the substrate, which are used for the fibers to channel alignment, finally, the device is sealed to further increase the alignment stability (Figure 3b) [33]. This device was obtained patterning on a glass substrate the grooves with a SU-8 photoresist by means of standard lithographic techniques, while the sealing was performed with a PDMS layer with a hole in the detection area, which is filled by index matching gel to allow for high quality image detection.

Newer approaches have also been presented to better match the device constrains, but still presenting a groove-based design with the disadvantage of a complicate multistep fabrication and the critical symmetrical fiber alignment [71,72,86,87,88]. Faigle et al. [71] presented a device based on the assembly of two asymmetrically etched glass substrates, which are subsequently bonded to each other with a thermal treatment. As reported in Figure 3c the bottom one contains shallow groves for fibers only, while the top layer hosts not only deeper fiber groves but also the sample channel. This design allows a perfect device functioning despite the two-layer misalignment that can occur during the bonding procedure. Other interesting works addressed the issue of the realization of disposable and mass producible devices for optical manipulation. Matteucci et al. [72] realized an easy to assemble Optical Stretcher chip in rigid Cyclic Olefin Copolymer (CoC) TOPAS 5013 by injection molding, exploiting the possibility to obtain grooves, and sample channel with different heights to achieve a symmetric fiber alignment (Figure 3d). De Coster et al. [87] instead presented a low-cost, disposable device realized in PMMA by double-side hot embossing. Despite potential design optimization, dual beam optical trap was efficiently proved.

Another approach that has been proposed to obviate to the difficult component alignment is presented by Cran-McGreehin et al. [89], which realized directly in a semiconductor laser material the microfluidic channel. Nevertheless, the limited laser powers of these sources may prevent the efficient use of this approach for particles stretching limiting it to the particle trap. A different method is the one that is presented by Osellame and coworkers, where, taking advantage from the capabilities of the fabrication technique, Femtosecond Laser Irradiation Followed by Chemical Etching (FLICE), a monolithic device for particle manipulation was realized. FLICE allows for easily creating within a single glass substrate both fluidic and optical elements (optical waveguides) [90,91], with the advantage of a precise and robust component alignment in a high quality substrate. Indeed, fused silica properties include solvent compatibility, excellent optical transparency, low auto-fluorescence, and high-pressure resistance. A first realization of a Monolithic Optical Stretcher (MOS) was performed integrating optical waveguides to a commercially available microfluidic device. In this way, a very robust platform was obtained that was characterized by a stable alignment of the two optical elements [70,92]. In a second device, not only the optical structures, but also the fluidic elements were obtained by FLICE, to enhance the precision of the component alignment [93]. The main drawback was related to the roughness of the channel, preventing high quality images, which are instead a major task for this application. A final device, which was presented by Yang et al. [73] with innovative irradiation geometry, was capable to address the roughness issue, giving rise to a very compact and efficient device for cell stretching and sorting. In Figure 3e, a picture of the final device with the corresponding microscope image is reported: the channel constituted by two inlets and two outlets is symmetrically intercepted by the optical waveguides (indicated by red traced line in the figure) were used to perform the optical manipulation.

### 3.2. Particles Movimentation

Different types of healthy, tumorigenic, and metastatic cells have been successfully measured with the OS, such as white and red blood cells [94], epithelial cells [33], and fibroblasts [95], revealing a certain heterogeneity in the populations. The correlation between the cell mechanical properties and functional changes in the sample highlighted the necessity to efficiently separate them on the basis of the mechanical properties, requiring the capability to move the target particles across the microfluidic channels. The sorting capability is therefore a necessary prerequisite to allow for further studies on a homogeneous subgroup with many applications in diagnostics and biology. Two distinct solutions were simultaneously presented in literature, both capable for the first time to implement an optical sorting driven by mechanical properties [71,73]. The difference between the two approaches lies mainly in the fabrication technique used: as previously discussed, Faigle et al. realized the device using a multistep lithographic approach, while Yang et al. realized a monolithic optofluidic device with FLICE. In both cases, the particles are first optically trapped and stretched, and subsequently sorted on the basis of the deformation analysis. The sorting is performed by simply unbalancing the laser power in the two arms of the optical trap, which gives rise to a net scattering force along the most intense beam propagation direction, pushing the particles towards one of the two sides of the channel. It is interesting to stress that to achieve a physical separation of the particles, it is necessary to optimize the microfluidic layout, adding multiple channel outlets, as shown in Figure 3e. The laminarity of the fluids that characterizes microfluidic flows, combined with the presence of multiple outlets, allows for the separation of particles that, flowing on different streamlines after the optical manipulation, can be efficiently separated.

**Figure 4:**
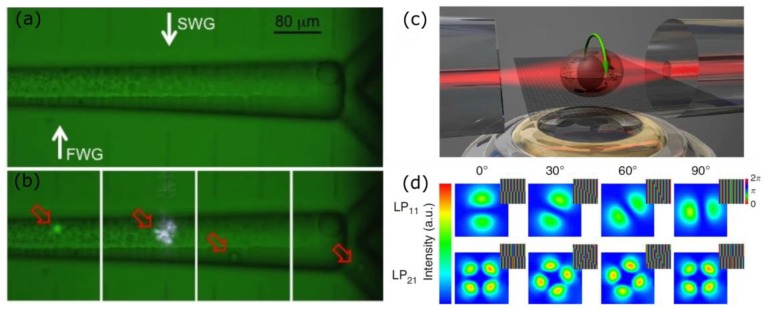
Panel (**a**) shows the microscope image of the integrated fluorescence activated cell sorter, presented by Bragheri et al. (reproduced from [97] with permission from the Royal Society of Chemistry). The waveguide used for fluorescence excitation (FWG) and the sorting waveguide (SWG) are highlighted by white arrows. The corresponding device validation is reported in panel (**b**). Panel (**c**) shows the scheme of an optical cell rotator, which using a non-rotationally symmetric beam is capable to rotate a particle. Panel (**d**) shows the different phase masks applied to the spatial light modulator and the corresponding supported mode at the fiber output. Changing the mask it is possible to rotate the mode and therefore the trapped specimen (reproduced with permission from [100]).

More, in general, other devices have been presented in literature where the optical sorting driven by different sample properties is performed with a single diverging beam that orthogonally faces a microfluidic channel. In 2008, Kim et al. presented the first version of an optical particle sorter that using an optical fiber coupled to a microfluidic channel performs a continuous sorting of the specimen flowing in the microfluidic channel on the basis of their dimension [96]. In 2010, Bragheri et al. presented an integrated fluorescence activated cell sorter, which was capable to optically sort particles on a fluorescence base [97]. In this paper, the authors took advantage from the capabilities of the FLICE technique to realize integrated waveguides that orthogonally face the microchannel for fluorescence excitation and sorting. Figure 4a,b shows the microscope image of the device and the corresponding device validation with biological samples. Martinez et al. used a similar approach in a device where the optical sorting was used to separate a subpopulation of cells from the main channel, forcing them to be processed in a microfluidic channel that suddenly narrows down. This microfluidic constriction chip was used to retrieve information on the sample mechanical properties [98]. Another device that exploits an unbalanced optical trap is the one presented by Yang et al. [99], who presented an integrated optofluidic chip that was capable of retrieving the viscosity of the fluid through experiments of optical shooting on single particles. The sample trajectory is recorded, analysed, and fitted with theoretical curves, allowing for the characterization of the fluid properties. 

Dual beam optical manipulation scheme has been successfully used also to perform particles rotation, which could be a very useful tool for a wide set of sample investigation techniques, such as refractive index analysis and single cell tomographic microscopy. Figure 4c shows a scheme of this approach that can be easily coupled with an external microscope objective for image acquisition. Optically induced particle rotation was first demonstrated with optical tweezers [101,102], but in this case, the imaging objective is the one also used for manipulation, thus it was limiting the field of view. In recent years, different approaches have been proposed in literature to induce sample rotation using a dual beam optical trap. Kreysig et al. [103] took advantage of an asymmetric dual beam trap, using on one side of the trap a multimode fiber that supports a non-rotationally symmetric beam. Therefore, using a mechanical rotator that turns the fiber, and hence, the supported mode, the specimen rotation is induced perpendicularly with respect to the beam propagation direction. Indeed, the reorientation of the cell in the trap tends to maximize the overlap between regions of high refractive index and areas of high field intensity. A second version of this method used adaptive optics to selectively excite different fiber modes, and it performed the specimen rotation with no need of any mechanical moving component [100]. In particular, using a Spatial Light Modulator (SLM) prior to the fiber coupling, they were capable of inducing optimized phase patterns to the beam wavefront and to obtain the desired rotationally asymmetric beam at the fiber output. Figure 4d shows the different phase masks that were applied to the Spatial Light Modulator and the corresponding supported modes at the fiber output. In 2012, Black and Mohanty presented the optical spanner based on a physical misalignment between the two fibers that induces the sample rotation [104], with the advantage that depending on the fiber offset, rotation could be induced in any direction. Subsequently, Kolb et al. got back to this idea with a dynamically tunable device [105]. Integrated pneumatic valves were used to induce an active control on the sample rotation. In this work, a dependence of the rotation rate on the inner structure and on the morphology of the sample was observed.

## 4. Evanescent Wave Optical Manipulation

When objects that are much smaller than the wavelength have to be manipulated and trapped, the use of laser beam impinging on them becomes less efficient. Indeed, for particles that are smaller than 100 nm, the optical forces acting on them become very weak [106,107]: in particular, the gradient force, which is responsible for the confinement of the particle in the plane that is perpendicular to the beam propagation is dependent from the third power of the particle radius. Focusing optics allow for the confinement of a laser beam to a spot whose size has a radius that is equal to λ/2NA (Abbe diffraction limit), where λ is the laser wavelength and NA is the numerical aperture of the lens. Given the typical range of wavelengths used for optical manipulation and commercial objectives NA ranging generally from 0.3 to no more than 1.4 for immersion objectives, it is not possible to obtain a beam waist smaller than a few hundreds of nanometers. With this limitation, it becomes evident how the decrease of the particle radius cannot be compensated by an equal increase of the electric field gradient just by tightening the focusing, since a ten-fold radius reduction, for example, from 1 µm to 100 nm, should correspond to 1000 times reduction of the laser beam spot size. The other free parameter is laser power, but increasing it could trigger heating effects that are generally not negligible, leading to thermal damage to the particles and/or surrounding liquid boiling.

A way to overcome this limitation lies in exploiting evanescent waves that can be generated in correspondence to the interface between two media. They are characterized by an imaginary wave vector in the direction perpendicular to the interface, which results in an exponential decay of the electric field without radiative losses (in absence of interaction with another object) in that direction. In certain conditions, there can also be a great electric field enhancement at the interface, due to the conservation of the normal component of the electric displacement field D (in absence of surface charge). The result is a very strong and confined electric field that is close to a surface, where the gradient is directed perpendicularly to it, causing a gradient force that can attract particles towards it. Since the first publication on particles moving along the surface of a waveguide in 1996 [108], many researchers demonstrated evanescent wave manipulation and trapping with a variety of structures. This approach, fundamental for very small particles, can also be applied to larger ones, as in many of the applications reported in the following sections where waveguides, photonic crystals, and plasmonic evanescent trapping methods are reviewed.

### 4.1. Waveguides

The paper of Kawata and Tani [108] first reported the trapping of both polystyrene (PS) and metallic particles of diameter between 0.5 and 5 µm on the surface of a waveguide and their motion along the propagation direction of the radiation inside the waveguide. Indeed, while the gradient force pulls towards the maximum of the electric field, which is located at the waveguide surface, the scattering force pushes the particles in the same way it does for conventional optical trapping, i.e., in the propagation direction of light. This means that particles can be manipulated but not trapped in a steady way, at least without a further effort. Nevertheless, this phenomenon is still useful, as it can be used to move very small particles inside a very confined region. A notable example of this technique, with a small variation, can be found in the paper of Yang et al. [109], where a slot waveguide has been chosen as a light confining structure, to attract and move PS particles with diameter down to 75 nm and λ-DNA molecules.

A similar effect was observed with the use of microfibers as waveguides. Frawley et al. [110] found that particles self-arrange along the fiber with two different equilibrium distances between them, while Irawati et al. [111] also observed a variation in the number of microspheres that are attached to the fiber related to particle and fiber size.

To further develop this technique and to obtain steadily trapped particles, different approaches have been adopted. One possibility, which is analogous to the case of conventional double-beam trapping, is the use of two counterpropagating beams, from the two ends of a single nanofiber [112], where the gradient force from the two beams sums up, while the two contributions of the scattering force have opposite sign. With this setup, the authors demonstrated the movements and halting of 713 nm diameter PS nanoparticles along a 910 nm diameter fiber in the two directions. Optical isolation of the fiber at the two ends is necessary in this case to avoid damaging the sources. Another example, with integrated optics, is the work of Hellesø et al. [113], where a waveguide is divided into two arms to form a ring with a gap in the middle. In this way, particles are transported along the guide, and steadily trapped in the gap thanks to the diverging beams. Here, the evanescent field is used to transport the particles, PS microspheres, and red blood cells, in proximity of the trap, and the scattering force generated by the radiation exiting from the guides keeps them in position. Another strategy to stop the motion of nanoparticles moving along a waveguide is to balance the gradient force imposed by the propagating electric field, with the drag force coming from the fluid in which the particles are suspended. Hu et al. [114] employed this method to trap and localize 700 nm PS spheres on the surface of a 3 µm diameter microfiber, with a fluid velocity of 6 µm/s compensating the scattering force that is induced by an optical power of 30 mW in the fiber. The gradient force can also have a contribution along the light propagation direction in the waveguide, if the field distribution changes in that direction. This is the case of tapered waveguides, discussed by Cai et al. [115], where an integrated waveguide sees a gradient force pointing in the opposite direction of light propagation in the region where it becomes narrower. The equilibrium points where particles can be trapped are the ones where gradient force and scattering force compensate themselves, and vary according to the taper geometry.

An interesting example of what can be achieved with this evanescent trapping in a waveguide is the microanalysis device that was proposed by Soltani et al. in 2014 [116]. Their optical circuit is composed by a base element that is referred to as nanophotonic standing-wave array trap (nSWAT), which is constituted by a directional coupler that splits the light injected in one coupler’s arm into the other arm; then, the two arms join forming a ring, giving rise to a standing wave. Each node of the standing wave is a trapping point for the particles to manipulate, and it can be moved along the ring by changing the phase in one of the two arms by means of a thermo-optical phase shifter. Combining more nSWATs on the same chip, the authors moved and sorted DNA molecules, with nanometric precision. In a later work, they presented a new version of their device [117], working at 1064 nm instead of 1550 nm, to reduce thermal effects, thus being able to increase laser power, and consequently trapping force.

### 4.2. Photonic Crystal Resonators

**Figure 5:**
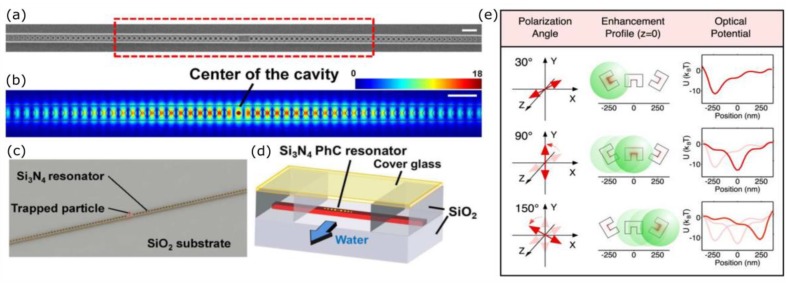
(**a**–**d**): One-dimensional (1-D) photonic crystal for particle trapping (Reprinted with permission from [119]. Copyright 2012 American Chemical Society: scanning electron microscope image of the resonant cavity (**a**), numeric simulation of the electric field intensity in the center of the cavity highlighted with a red dashed rectangle in the panel (**b**), schematics of the setup for trapping experiments (**c**,**d**). (**e**) Drawing illustrating how the rotation of electric field polarization results in the excitation of the structure aligned perpendicularly to it, moving the PS sphere towards the position of minimum optical potential (Reprinted with permission from [121]. Copyright 2014 American Chemical Society).

Waveguides are not the only devices able to generate evanescent waves in a microfluidic chip. Photonic crystals are structures with a periodic refractive index variation, which can allow or prevent light propagation with a very high sensitivity to the wavelength. Photonic crystal resonators can be designed so that light is confined in a very small region, even comparable with the resonant wavelength, with a notable electric field enhancement. The simplest version of such a device is a one-dimensional (1-D) resonator, which resembles a waveguide with periodic or quasi-periodic features. In the work of Mandal et al. [118], the resonator is excited by a waveguide that is parallel to it by evanescent coupling. A series of equally spaced and aligned holes selects the resonance wavelength, and a wider gap in the middle of the resonator creates the region with higher field intensity, which acts as an optical trap. The authors demonstrated the trapping of 48 nm and 62 nm PS particles, as well as 500 nm ones, also showing how detuning the laser from the resonant wavelength allowed for the release of the particles trapped in the photonic crystal, and their capture and transport by the excitation waveguide, whose trapping efficiency is not so sensitive to the guided wavelength. A photonic crystal can also be integrated directly into a waveguide, as in the papers of Chen et al. [119] and Renaut et al. [120]. In the first case, a series of holes with fixed periodicity was present in the waveguide, and in the gap that was left in the middle of the resonator, a smaller hole was introduced to increase field intensity in that point, as shown in Figure 5a–d. The device was validated with proteins, quantum dots, and 22 nm nanoparticles. According to the authors, the main advantage of this structure, besides the ability of trapping nanometric objects, is the very low temperature increase that is given by the absorption from the samples. This aspect is important, since even a small temperature rise next to the trap could trigger thermophoretic effects that would cause particles to move in the opposite direction of the thermal gradient, thus escaping from the trap. Higher temperature could also damage the samples. In the second work, a similar structure is presented, with just a larger gap in the middle, and the authors employed it to accumulate and aggregate nanoparticles, activating the trap with about 300 µW power, which is notably low when compared to the several mW used in the previously cited papers.

Besides trapping, the rotation of a particle is possible with photonic crystals. An example is presented in the paper by Kang et al. [122], in which carbon nanotubes and biological microtubules are oriented, following the electric field of the TE mode, which was guided in the structure described in ref [118]. With just the electric field, the rods can be aligned in a fixed position, with a time constant that is determined by the viscosity of the surrounding fluid. Then, starting a flow orthogonal to the resonator, the rods, which are preferentially trapped from one of their extremities, are subject to a torque that provokes a rotation, until hydrodynamic forces are balanced by the optical ones.

1-D photonic crystals are not the only possibility for optical manipulation: also, two-dimensional (2-D) structures have been proposed by researchers. The insertion of small slots inside a rectangular photonic crystal lattice, known as Suzuki-Phase lattice, has been suggested by Ma et al. [123], in order to increase the quality factor of the cavity and to tune the resonant wavelength. The trapping of bacteria using a 2-D photonic crystal has been experimentally demonstrated by Leest and Caro [124], who tested three different lattices, which were created by inserting different defects in the center of a periodic structure, excited by a nearby waveguide.

### 4.3. Plasmonic Traps

A last way to finely control and to generate local electric field confinement relies in the use of plasmonic resonances. The field enhancement provided by collective electronic oscillations on a metallic surface, known as surface plasmons, can be used to provide a very confined and powerful optical trap. These oscillations can be excited on a flat surface by an evanescent wave, and propagate along it, while having an exponential decaying component at both sides of the interface, as opposite to evanescent waves. In this first case, they are known as Surface Plasmon Polaritons (SPPs). When the metallic interface is very localized, as in metallic nanoparticles, the plasmonic oscillation can arise upon illumination by a regular light beam, and it is called Localized Surface Plasmon (LSP). With this second type of plasmonic oscillation, there will be no energy propagation along the surface, hence the name LSP.

SPPs will transfer momentum to the objects nearby the surface, in a similar fashion to evanescent waves on dielectric waveguides, but with higher efficiency. Cuche et al. showed how they could propel gold nanoparticles through the force that is imposed by the SPPs that were generated on a gold film deposited on a glass prism [125]. This plasmon excitation technique relies on the energy transfer between the evanescent wave excited on the glass-gold interface and the plasmonic mode on the other gold surface. The researchers used two different wavelengths to excite the SPPs, and showed that they were able to separate two populations of 50 and 125 nm radius, matching the two corresponding LSP resonance frequencies. This work takes advantage from the fact that planar plasmonic structures can support a broader range of wavelengths than the nanoparticles.

Many trap geometries are possible when localized traps with nanometric size metallic structures are considered. Kang et al. [126] fabricated an array of gold diabolo shaped nanoantennas and used them to trap PS nanoparticles in water and silica nanoparticles in oil. They calculated that the maximum of the electric field, during excitation with a beam that was perpendicular to the substrate, was on the edge of the diabolo structure. Experimentally, they showed how PS nanoparticles accumulated around the structures, i.e., in correspondence with the maximum position, while the silica particles were found in the center of the antennas, in the electric field local minimum. This happened since the silica refractive index is smaller than the one of the oil, so the gradient forces pushed them away from the maximum of the electric field. Another example is the work of Sergides et al. [127], where the authors used connected ring gaps that were dug in a 50 nm gold nanolayer as trapping sites. They used an excitation system in the Kretschmann configuration, to simultaneously target all of the trapping regions, and showed that, while all the nanoring arrays were attracting 100 nm diameter PS nanoparticles, the amount of particles trapped varied for the different gap sizes. The detection of trapped nanoparticles was performed with great precision acquiring extinction, reflection, and ellipsometric spectra on the nanostructures.

Not only trapping, but also the nanoparticles manipulation has been demonstrated with devices based on LSPs. Zheng et al. [121] presented a C-shaped engraving with the field enhancement localized in the internal part of the C, which can be excited only by light polarized in the direction perpendicular to the C. They placed a series of engravings tilted one with respect to the other, and rotating the excitation beam polarization, it was possible to move along the row of nanostructures the maximum of the electric field, and consequently a nanoparticle, as depicted in Figure 5e.

As a last example of application of plasmonic devices, placing a plasmonic nanostructure on top of a photonic crystal resonator can help to enhance the performance of the cavity as a very confined trap with ultra-low excitation power, as shown by Conteduca et al. [128]. The authors realized a gold bow-tie shaped nanoantenna on a 1-D resonator, showing the ability of trapping 100 nm PS and gold nanoparticles, and also exploiting the different shifts of the resonator’s peak due to the different kind of particles, in order to detect the trapping.

## 5. Conclusions

Manipulation of single particles is quite a hot topic, as demonstrated by the ample literature in which different methods are proposed. These include the use of magnetic, acoustic, or electric fields, but also droplet based manipulation or fluidic forces. All of these techniques show pros and cons, and could be preferred on the basis of the specific application. In this work, we decided to restrict the discussion to optical based manipulation techniques, presenting the properties of this approach. In particular, we have summarized the ample literature on optical manipulation, focusing on the synergistic combination of optical forces and microfluidics, which can be used to increase the throughput and the measurements automation.

As thoroughly discussed, many groups are currently following this line, presenting compact devices that optically investigate the sample flowing in microfluidic channels. We believe that the next step will concern the simplification of the actual approaches, creating even more compact and portable platforms, easy to use also for non-expert-end-users. The other fundamental crux is related to the throughput, indeed the actual value can be a limit for certain applications where large populations have to be analyzed and to address this aspect new solutions have to be found. Nevertheless, for many other applications, optical manipulation represents the ideal tool. Indeed, at the current state of the art, this is the ideal method to perform a careful investigation of rare cells or small sample populations, where the measurement precision, the single cell specificity, and the possibility to collect the entire population or part of it, after the analysis are mandatory.
